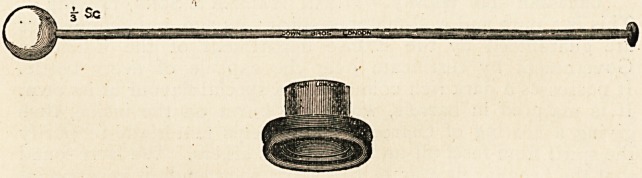# Notes on Preparations for the Sick

**Published:** 1897-03

**Authors:** 


### Notes on Preparations for the Sick.

---

**Ferratin.** — BOEHRINGER & SOEHNE, Mannheim. — This is described as the ferruginous element of food. It is a fine powder of reddish-brown colour, without odour or taste, containing about seven per cent. of iron. It is artificially prepared from egg-albumen and iron salts in alkaline solution; it is not a mechanical mixture, but a chemical combination of iron and albumen, resembling in all respects that which can be obtained from the liver of the pig, and which is looked upon by Schmiedeberg as the natural form in which iron is taken in food. In an exhaustive clinical investigation of the action of Ferratin as a drug it has been found to give encouraging results, and has shown itself to be worthy of trial for all those cases where the ordinary iron salts do not at once accomplish the end in view. Twenty to thirty grains may be given daily.

---

**Tabloids: Lithia Bitartrate,** gr. v.; **Lithia Citrate,** gr. iv.; **Cerium Oxalate,** gr. v.; **Erythrol Tetra-Nitrate,** gr. j.; **Caffeine Comp.,** gr. iv. (caffeine gr. j., antipyrin gr. iij.); **Phenacetin Comp.** (phenacetin gr. iv., caffeine gr. j.); **Cascara and Belladonna; Piperazine,** gr. v. (uric acid solvent); **Uranium Nitrate,** gr. j. (for diabetes); **Residuum Rubrum,** gr. v.; **Chloralamid and Bromide of Potassium,** āā. gr. v.; **Red Gum** (eucalyptus rostrata); **Quinine Salicylate,** gr. iij.; **Colchicum Compound** (acetic extract of colchicum, gr. j., salicylic acid, gr. iss.); **Blue Pill and Compound Rhubarb** (blue pill gr. iiss., comp. rh. pill gr. iiss.); **Iridin Compound** (iridin gr. ij., ext. hyoscy. gr. $\frac{1}{2}$, comp. rh. pill gr. iss.); **Mercury with Chalk and Opium** (mercury with chalk gr. j., opium powder gr. $\frac{1}{6}$); **Pepsin, Bismuth, and Strychnine** (pepsin gr. j., bism. subcarb. gr. iij., strych. sulphate gr. $\frac{1}{100}$); **Pepsin with Strychnine** (pepsin gr. j., strych. sulph. gr. $\frac{1}{100}$); **Trional,** gr. v.; **Tetronal,** gr. v.; **Bromide Compound** (strontium bromide gr. ij., sodium bromide gr. ij., ammon. bromide gr. j., sodium arseniate gr. $\frac{1}{60}$); **Codeine,** gr. $\frac{1}{2}$; **Saxin** (600 times sweeter than sugar), gr. $\frac{1}{4}$.—BURROUGHS, WELLCOME & Co., London.—These additions to the now long list of Tabloids have a range of utility, which is for the most part indicated by their composition and their description. The **Residuum Rubrum** are prepared from the carefully dried red residuum of venous or arterial blood. They have been found useful in osteo-arthritis, given in doses of twelve five-grain tabloids daily. The liquid preparations of Sanguis are very distasteful to most patients, but we see no reason why the ½-gr. tabloids should not be taken freely by those patients who can easily swallow pills. The **compound tabloids** appear to be excellent and useful combinations of their various ingredients.

---

**Nutroa Food.**—NUTROA LIMITED, London.—A comparison of the chemical composition of this food shows that it does not materially differ from the "ideal diet for children" given by Dr. Halliburton. The proteids amount to 19.64 per cent., the carbo-hydrates to 67.02 per cent., and the fats to 12.12 per cent. It is a cooked, malted food, enriched by the addition of proteids, and therefore it contains within itself in an easily-digested form all the elements of a sufficient diet. These compound foods may be perfect in theory as a simple but sufficient diet, but we have yet to find the patients who will submit to live on a repetition of the dose of such foods without variation. It is necessary that foods should be agreeable to the palate as well as that they should have a satisfactory chemical composition, and hence there is the less need that any one food should have the normal proportion of proteids of an ideal diet. Dr. Halliburton informs us (*Brit. M. J.*, 1896, i. 563) that he has not authorised the use of his name in connection with this food, as "the ideal diet for children," and that he does not consider it to be in any sense a substitute for human milk. Nevertheless it is clear that a food of this composition must often be useful, and that its nutrient value is far in excess of the ordinary farinaceous foods in daily use.

---

**Kreochyle, Barff & Wire's Liquid Meat.**—THE KREOCHYLE Co., London.—The promoters claim for this "liquid meat that it is in every sense of the word a food, containing the whole of the soluble albumen in an uncoagulated condition, which can be digested by the weakest stomach. It is also a powerful stimulant, containing, as it does, the whole meat extractives." We do not question either of these statements; but nevertheless we cannot but combat the idea that these fluids are so concentrated as the promoters would have us believe. When we are informed that the intestine of a turkey filled with Kreochyle and suspended in water discharged 90 to 92.5 per cent. of its albumen into the surrounding water, we naturally infer that the amount of albumen is large and the fact an important one, and hence it is of interest to enquire how much albumen the fluid contains. The picric acid tube answers this question by showing that the amount of albumen (0.1 per cent.) is much less than is often contained in ordinary albuminous urine of a minor grade, and which is discharged daily by the pint, whereas the ordinary dose of the liquid meat is only a fraction of an ounce. The fluid has a gravity of 1024, and gives the hæmoglobin bands with the spectroscope. The hæmoglobin and extractives have a physiological value both as food and as stimulant, but that value may be easily over-estimated, and the term liquid meat conduces to this end. Patients take this fluid freely and without complaint; it has no positive objectionable qualities.

---

**Champagne.**—CHARLES HEIDSIECK, Reims.—Till we received samples of this wine we thought there was only one Heidsieck of "Dry Monopole" fame; but it seems from a pamphlet sent with the wine that the only Heidsieck of Reims is the firm bearing the trade-mark of this brand. The pamphlet gives a clear account of the processes through which the wine goes before it results in the form familiar to us.

---

**Canadian Club Whisky.**—HIRAM WALKER & SONS, London.—This is a pure and well-matured spirit; its age and genuineness are guaranteed by the Excise department of the Canadian Government by certificate over the capsule of every bottle. It possesses a dark rich colour, and a special flavour of its own. It is matured in barrels, which are burnt on the inside, thus giving a coating of charcoal, which helps materially to purify the spirit from fusel oil and deleterious ethers. We have found that the peculiar flavour of this spirit soon lends it an especial charm.

---

**Chinosol.**—B. KÜHN, London.—This is a new antiseptic, which is non-poisonous and non-corrosive. It does not coagulate albumen, and is freely soluble in water. We have tried it and found it pleasant to use, and without the disagreeable odour of many antiseptics.

---

**Lung Gymnastics.**—The use of an improved breathing-tube for the inhalation and exhalation of common air is advocated by the Hygienic Supply Company, of Boston, Mass. The tube, as we infer from the description of what we have not seen, is arranged to give increased resistance in exhalation, whereas the inhalation is not impeded; accordingly it tends to develop the respiratory muscles and increases the capacity of the lungs. The tube is to be used for five or ten minutes continuously three times a day, gradually increasing to half-an-hour thrice daily. When you can do this easily with the small valve "you may be sure you are on the road to good health." This is the statement of the promoters, but we must refrain from expressing any opinion on this proposal to improve the normal form of the respiratory channels.

---

**A New Pleximeter.**—Down Bros., London.—In the Report on Medicine last September attention was drawn to a hammer suggested by Dr. Kingscote, of Salisbury, for the purposes of combined auscultation and percussion. Dr. Kingscote says:

"Ever since 1761, when percussion as a means of physical diagnosis was first suggested by Avenbrugger of Vienna, it has been generally recognised that deep percussion of the thorax is not free from uncertainties arising—(1) from the vibrations of the chest-wall veiling the note really given out by the structure underlying the plessor, and (2) from the fact that percussion, when sufficiently hard to produce a true note, is apt to hurt the patient. I have devised a pleximeter which overcomes all these difficulties. It consists of a wooden cone, made with a shoulder to which a thick indiarubber ring is attached in such a manner that when the instrument is firmly pressed against the chest the apex of the cone touches a definite point on the skin in the centre of the ring. If the other end of the cone be now struck smartly with a hammer having a flexible handle, the true note of the underlying structure is elicited. The fact that experiments have been repeated many times with invariably uniform results proves the possibility of obtaining an accurate definition of the boundaries of the heart even when it is overlapped by lung tissue. The dulness shown by the pleximeter is always somewhat external to that elicited by finger percussion. A special form of hammer is used with the pleximeter, its head being made of lamb's-wool so compressed that it cannot be beaten out of shape—a necessary condition for the production of an absolutely true note, and it is of such a weight that the required note is obtained without the absorptive power of the surrounding ring being overcome. The finger may, however, be employed as a hammer with equally good results when the wrist is very flexible. In using the pleximeter it must be applied firmly to the region to be percussed, and there must be no alteration in its position when it is struck with the hammer."

## Figures and Tables

**Figure f1:**